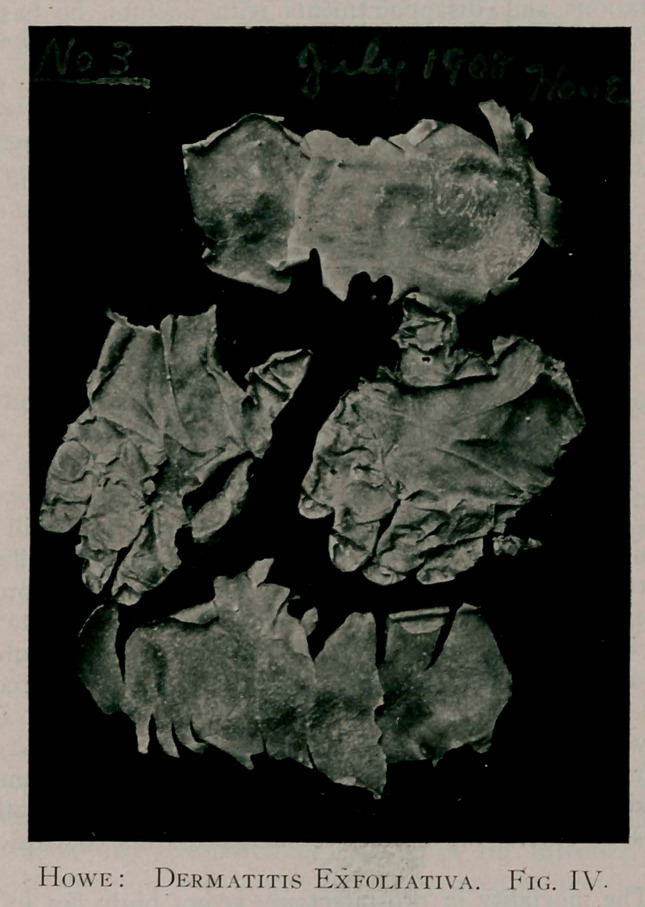# An Interesting Case of Dermatitis Exfoliativa

**Published:** 1909-07

**Authors:** William A. Howe

**Affiliations:** Phelps, N. Y.


### An Interesting Case of Dermatitis Exfoliativa

By WILLIAM A. HOWE, M. D. Phelps, N. Y.

THE case which I am desirous of bringing to your attention today, had its inception in March, 1895. It again manifested itself in January, 1900. These two attacks were incorporated in an article which I was privileged to present to this society on October 16, 1900, and were subsequently published in the Buffalo Medical Journal in February, 1901. Since January, 1900, the same patient has suffered two further attacks, one in December, 1907, and the other in July, 1908.

It is these recent relapses which have induced me to supplement my former report, and still further record my latest experience with this rare and highly interesting condition.

The symptoms from which this patient suffered differed more in degree than in character. In the first two attacks he was profoundly ill, in the third he remained in bed only a few days, while in the fourth he scarcely kept the house.

Inasmuch as his local and general symptoms were so fully set forth in my former article, to which reference has been made, and since you will find the symptomatology of this disease comprehensively discussed in our more recent textbooks on dermatology, it seems rather unnecessary for me to repeat them here, thereby needlessly prolonging this report. Notwithstanding the wide range of constitutional and local symptoms, which he exhibited, the characteristic desquamation of the epidermis was in each instance much the same in its extent.

Not so, however, in the damage done to the deeper tissues. Here the more intense the dermic inflammation and the longer its duration, the more serious and lasting was the damage done to the subepidermal tissues. In spite of the fact that for thirteen years he has been a periodical sufferer from this strange malady, his skin, while rather dryer and rougher than that of the average individual, is far from the condition in which you would naturally expect to find it. Between these relapsing attacks he apparently enjoys the best of health.

One subjective symptom, not mentioned, so far as I know, in any of our textbooks, has been that of gastro-intestinal fermentation, accompanied with a great deal of flatus. While not wishing to attempt the etiological consideration of this disease, the uniformity and persistency of this prodromic symptom, in my case, has been such that I should not be justified in omitting its mention. Possibly it is simply coincident, and yet it may, at least be suggestive of an autointoxic condition, most potent in its bearing, to the ultimate solution of the much disputed cause of this obscure affection.

HOWE: DERMATITIS EXFOLIATIVA. FIG. I.

But this case has appealed to my interest, not so much on account of its subjective or general symptoms, as from the rare specimens, which have been collected throughout its course.

The specimen marked No. 1, was taken from this patient in January, 1900. It is possible that some of you may recognise it as being one of those which I exhibited in Rochester, when I first presented this case. Since then, it has been kept in an ordinary paste board box, and though more than eight years have elapsed, it is, as you will see, in a perfect if not a permanent state of preservation. This has evidently arisen from the completely desiccated condition of the epidermis when it was exfoliated.

While you will notice that only one specimen is shown from this attack, you will further observe from the photographs marked No. 1, 1900, that both hands and both feet were equally involved.

HOWE: DERMATITIS EXFOLIATIVA. FIG. II.

The specimens marked No. 2, were secured on December 13, 1907. Like those of 1900, they are very complete and apparently in a lasting state of preservation. At this time, the epidermis of the right hand was turned inside out in its removal, much as you would remove a glove in the same manner.

The exhibit marked No. 3, resulted from the relapsing exfoliative dermatitis from which he suffered in July, 1908. This last recurrent exacerbation, as you will note, followed the preceding one in eight months, for which reason the desquamated epidermis was much thinner and softer than that seen in the earlier attacks.

These last specimens when given off retained for several weeks an appreciable sense of moisture, yet they have never shown any disposition to disintegration and are now in the same fixed state of preservation so characteristic of the earlier ones.

While dermatitis exfoliativa or pityriasis rubra may be comparatively frequent in the experience of the busy dermatologist, I can but believe it is extremely rare to the general practition-

HOWE: DERMATITIS EXFOLIATIVA. FIG. III.

er. Except one acute and rapidly fatal case this has been the only instance in which it has come under my observation in over twenty years of rather extensive general practice. What has been the experience of my hearers? How many of you have seen a case like it and what was your experience with it? In searching medical literature I fail, especially in the several journals at my disposal to find recorded any cases of this strange disease, which has in its existence so many possibilities.

During my patient's last illness, he was given a newspaper clipping, with an account of a similar case in Trenton, N. J. Upon his urgent request and to satisfy my own desire, I wrote to the gentleman whose name was given in the paper. Within a few days thereafter I received from him a most interesting account of his sickness. While not at liberty to use this gentleman's name, I can, with no violation of trust, briefly quote his case. I am moved to do this that one more case may find its way on record, hoping thereby to arouse a little extra interest in the study of this

HOWE: DERMATITIS EXFOLIATIVA. FIG. IV.

malady, which is not only so mysterious in its etiology but, thus far, absolutely unyielding to any known method of treatment.

CASE NO. III.—W. U. C., male. While his mother told him he had it when a child his first recollection of having had it was in 1867. It did not trouble him again until 1879, since which time he has had eight attacks, varying from three to eight years apart. Its advent is generally ushered in with a chill, after which his hands and feet begin to swell, and his whole body turns a deep red. This redness lasts from six to forty-eight hours, after which an intense itching comes on and lasts until the skin of his hands and feet comes off. Extreme prostration is another symptom from which he complains. He further claims that quinine sulphate, grs. iv., or a small dose of belladonna, will excite an attack at any time. No other member of his family, nor any of his grandchildren have ever been afflicted with it, and he is positive that there is nothing about it that is in any way contagious. He further adds that after more than forty years of experimentations and disappointments with doctors, he has been forced to believe that none of them know anything about the disease or any relief for it.

He kindly enclosed a specimen of epidermis, which was removed during one of his attacks. This you will find among my other exhibits, so marked as to identify it from those of my other cases.

67 E. Main Street.

## Figures and Tables

**Fig. I. f1:**
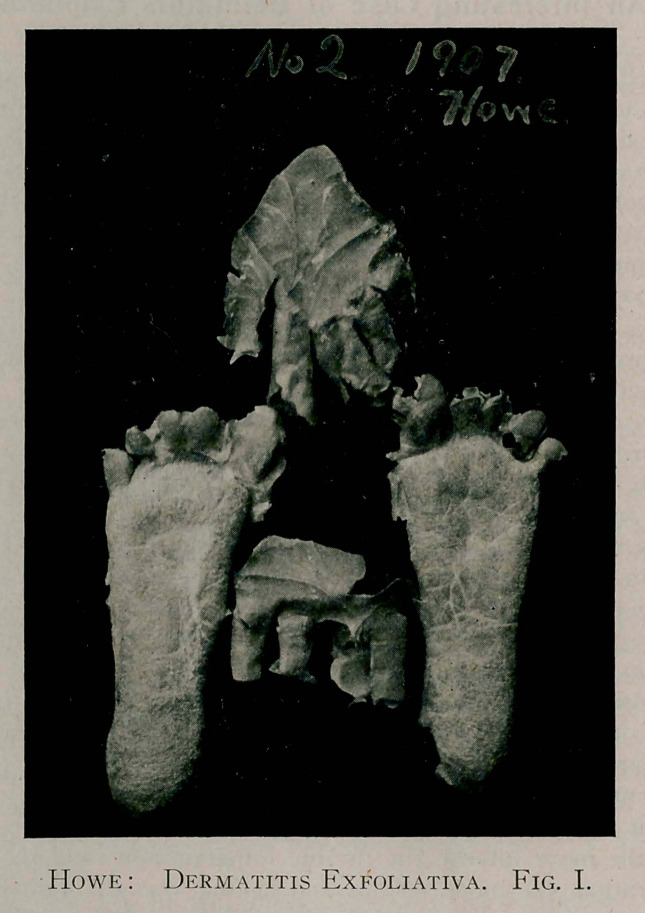


**Fig. II. f2:**
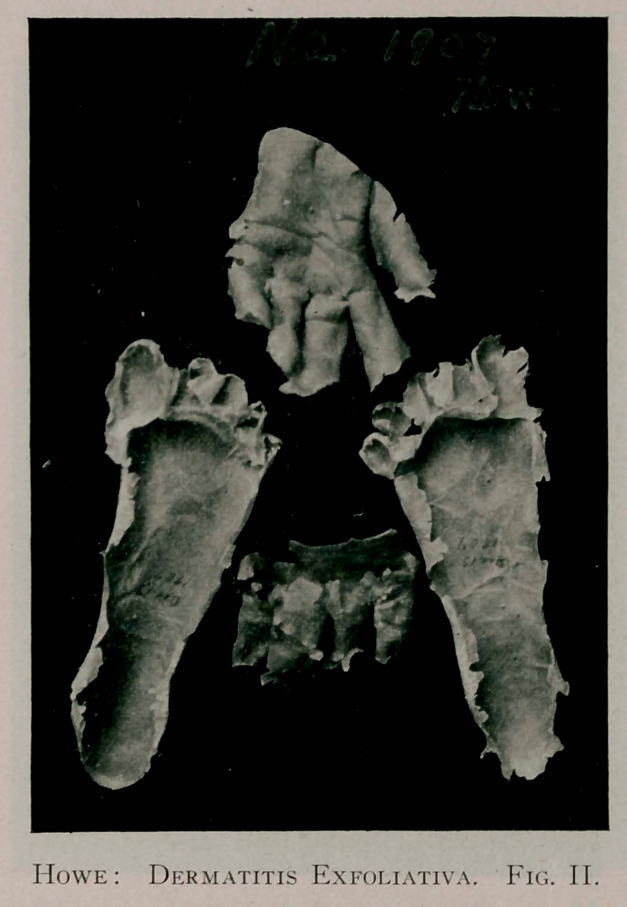


**Fig. III. f3:**
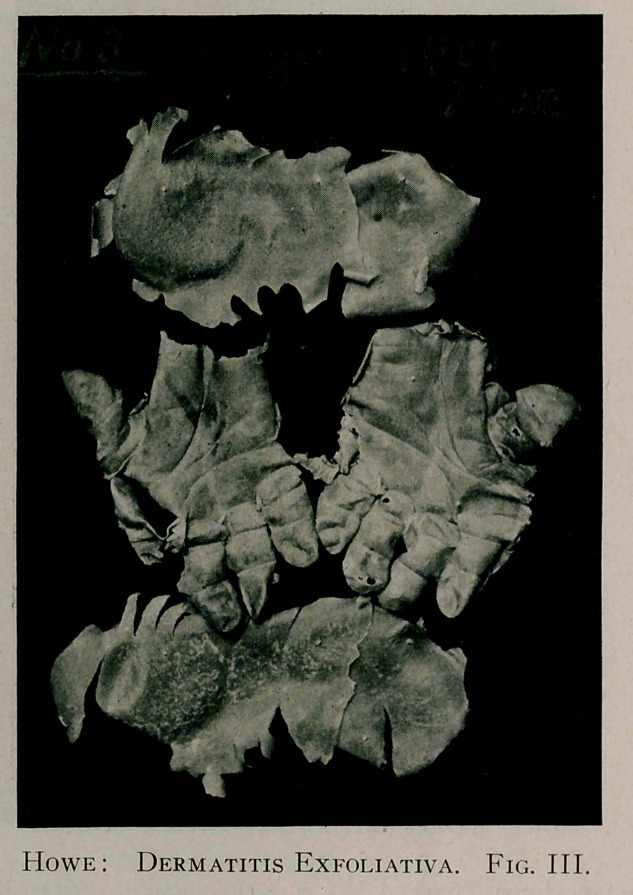


**Fig. IV. f4:**